# An Unusual Case of Cavitary Lung Lesion and a Brief Review of Literature

**DOI:** 10.7759/cureus.9361

**Published:** 2020-07-23

**Authors:** Kishor Khanal, Xuan Guan, Parmatma Parajuli, Manoucher Manoucheri

**Affiliations:** 1 Internal Medicine, AdventHealth, Orlando, USA

**Keywords:** cavitary lung lesion, actinomycosis, pulmonary embolism, pulmonary infarction, lobectomy

## Abstract

Large cavitary pulmonary infarctions are extremely rare. Here, we report an unusual case of large pulmonary infarction due to pulmonary embolism (PE). This was complicated by secondary infection with Actinomyces leading to cavitary abscess formation. The patient had minimal non-specific symptoms despite extensive involvement and was treated with lobectomy, antibiotics, and anticoagulation, with expedited recovery. This case depicts a rare association between PE/pulmonary infarction and pulmonary actinomycosis. Furthermore, it highlights a high index of suspicion needed to diagnose these two conditions in low-risk individuals without traditional risk factors.

## Introduction

Non-malignant cavitary lesions may mimic malignant lung lesions on most of the radiographic modalities including thoracic computed tomography (CT). Primary lung malignancies can be detected in as high as one-fifth of CT of the thorax when presenting as cavitary lesions. The remaining etiologies may be due to bacterial, parasitic, and invasive fungal infections, as well as granulomatosis with polyangiitis, sarcoidosis, venous thromboembolism (VTE), and lung metastasis from extra-pulmonary primaries [1]. Cavitary lung lesions of various etiologies may be encountered in patients and usually present with respiratory symptoms. Pulmonary embolism (PE) is a noninfectious cause of a cavitary pulmonary process. PE causes infarction in fewer than 15% of cases, and only about 5% of infarctions cavitate [2]. Virchow’s triad suggests that the pathogenesis of venous thrombosis is damage to the vessel wall, disorders of coagulation, and stagnant flow. Damage to the vessel wall can be due to infection, infiltration, or trauma. However, more common problems are coagulation defects that cause a hypercoagulable state [1].

Actinomycosis is a rare chronic disease caused by *Actinomyces* species, an anaerobic gram-positive bacterium that normally colonizes the human oral cavity, gastrointestinal tract, and genital tract [3]. The regions mostly involved are the cervicofascial area, the thorax, and the abdomen. The thoracic variety accounts for approximately 15% of the cases, where clinical pictures of pulmonary neoplasm, abscess, and empyema have been described [4]. Diagnosis of actinomycosis is frequently difficult because it often infects pre-existing cavitary disease in the lung. Consequently, the infection may progress to the stage where it will not respond to medical treatment alone. In such cases, surgery provides the best approach to definitive diagnosis and treatment [5]. To the best of our knowledge, this is the first reported case of a large pulmonary infarction complicated by *Actinomyces* *odontolyticus* infection in the absence of any other risk factor.

## Case presentation

A 64-year-old otherwise healthy athletic male presented with complaints of fatigue, low-grade fever, and mild non-productive cough for two months. He did not have a significant past medical history. He was a non-smoker and used to drink alcohol socially. The patient recalled right calf pain about six months prior to the presentation due to “pulled muscle” when he jumped while watching a basketball game, which resolved on its own. He had mild right shoulder, upper back, and right-sided pleuritic chest pain a few months ago for which he saw a chiropractor and the symptoms improved. Family history was positive for lung cancer in his father and uncle. Screening colonoscopy performed three years ago was normal. He had no recent sick contacts or significant travel history.

The patient saw his primary care physician for unremitting low-grade fever and was referred to our facility for abnormal chest X-ray findings. Vital signs were significant only for a low-grade temperature of 99.5°F. Physical examination revealed the absence of breath sounds on the right lung base. Initial labs were significant only for elevated erythrocyte sedimentation rate (ESR), C-reactive protein (CRP), and anemia (Table 1). Electrocardiogram (EKG) was unremarkable.

**Table 1:** Clinical laboratory results

Measure	Reference Range	Admission Lab	Interpretation
White cell count (per μL)	4,400-10,500	7,800	Normal
Red cell count (per μL)	3,750,000-5,000,000	4,190,000	Normal
Absolute neutrophil count (per μL)	1,500-7,500	5,590	Normal
Absolute lymphocyte count (per μL)	1,000-4,800	1,030	Normal
Platelet count (per μL)	139,000-361,000	494,000	High
Hemoglobin (g/dL)	11.4-14.7	10.8	Low
Hematocrit (%)	34.3-45.5	34.3	Low
Mean corpuscular volume (fL)	82.4-99.3	81.9	Low
Sodium (mmol/L)	135-145	134	Low
Potassium (mmol/L)	3.5-5.0	4.0	Normal
Chloride (mmol/L)	98-110	97	Low
Calcium (mmol/L)	8.5-10.5	8.8	Normal
Carbon dioxide (mmol/L)	24-32	23	Low
Anion gap (mmol/L)	5-15	14	Normal
Glucose (mmol/L)	70-100	101	High
Blood urea nitrogen (mg/dL)	5-25	14	Normal
Creatinine (mg/dL)	0.6-1.2	0.93	Normal
Total protein (g/dL)	6.5-8.0	7.8	Normal
Albumin (g/dL)	3.2-5.5	3.6	Normal
Total bilirubin (mg/dL)	0.1-1.5	0.2	Normal
Alanine transferase (units/L)	4-51	47	Normal
Aspartate transferase (units/L)	5-46	35	Normal
Alkaline phosphatase (U/L)	40-129	122	Normal
Lactate dehydrogenase (U/L)	60-200	187	Normal
Prothrombin time (seconds)	11.5-14.9	13.9	Normal
International normalized ratio	0.8-1.2	1.10	Normal
Activated partial thromboplastin time (seconds)	22.0-38.0	30.7	Normal
C-reactive protein, inflammatory (mg/L)	<5	66.7	High
Erythrocyte sedimentation rate (mm/hour)	0-20	99	High
Lactic acid level (mmol/L)	0.5-1.9	0.6	Normal
Procalcitonin (ng/mL)	<0.10	0.08	Normal
Ferritin (ng/mL)	24-336	988	High
Iron level (μg/dL)	45-182	30	Low
Total iron binding capacity (μg/dL)	221-481	207	Low
Iron saturation (%)	30-44	14	Low

**Figure 1:**
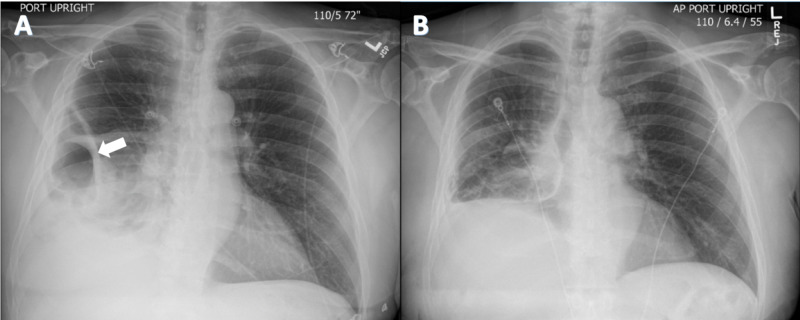
Chest X-ray. (A) Chest X-ray on presentation showed loculated fluid collection (arrow) with associated atelectasis at the right lung base. (B) Postoperative interval improvement in aeration of the right lung base. Right hemi-diaphragm remained persistently elevated with trace right pleural effusion and adjacent atelectasis.

**Figure 2:**
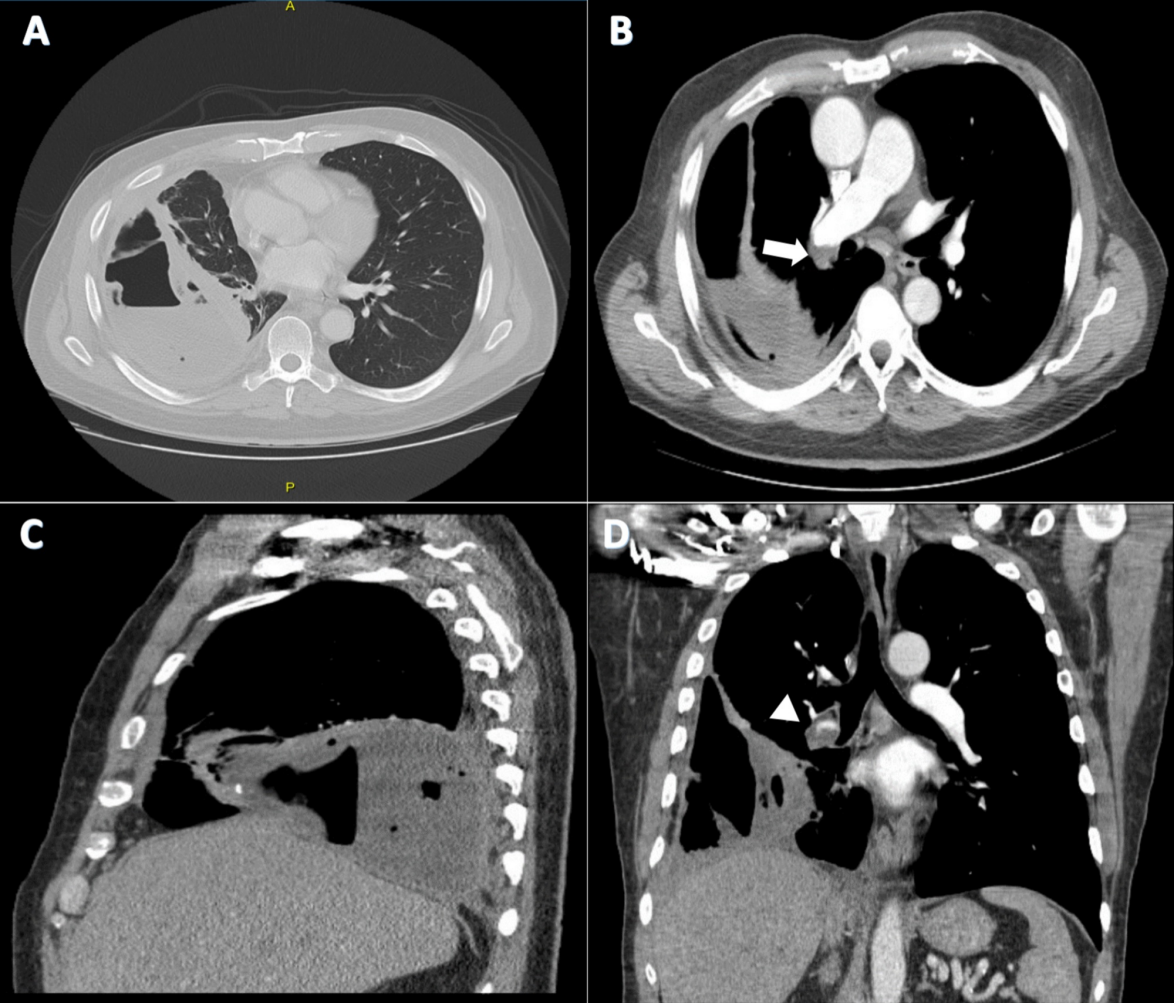
Chest CT with contrast with sagittal and coronal reconstruction views. (A) Axial lung window reveals walled-off collection with air-fluid level in the right lower chest. (B) Distal right main pulmonary artery occluded by pulmonary embolus can be seen (arrow). Sagittal view (C) highlights the collection described above and coronal view (D) highlights the collection as well as the pulmonary embolus in the right pulmonary artery (triangle).

Chest X-ray revealed loculated fluid collection in the right lung base with atelectasis (Figure 1A). CT chest with contrast revealed PE occluding the distal right main pulmonary artery (Figure 2). Some components of heart strain including flattened septum were present. A walled-off collection measuring 18.0 x 7.6 x 9.5 cm with air-fluid level was present in the right lower chest. CT of the abdomen and pelvis with contrast was unremarkable except for gallstones. He was found to have acute deep vein thrombosis (DVT) in the right lower extremity, noted in the middle to distal femoral and popliteal veins on Doppler ultrasound. The patient was started on heparin infusion and empiric intravenous piperacillin-tazobactam. Transthoracic echocardiogram was normal with no evidence of right heart strain. Bronchoscopy showed no endobronchial lesions. Copious secretions were noted from the right middle and lower lobe, which were suctioned. Infectious etiology workup including acid-fast bacteria (AFB) and Legionella cultures, *Pneumocystis jiroveci* gram stain, *Mycoplasma pneumoniae* IgM (immunoglobulin M), and HIV screening were all negative.

**Figure 3:**
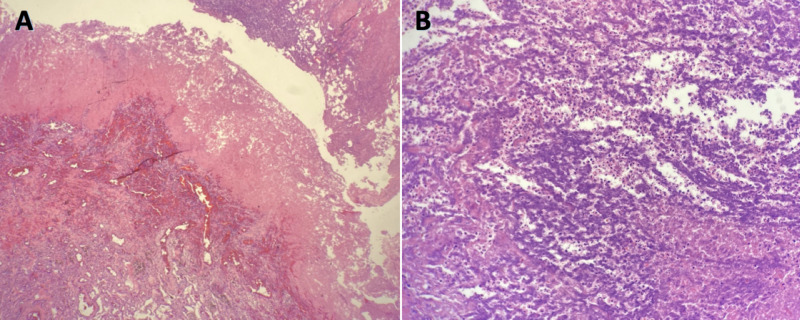
Lung biopsy showing benign pulmonary parenchyma with extensive necrosis, bronchopneumonia, and marked abscess formation with original magnification x40 (A) and x200 (B).

Based on the nature and extent of the lesion, cardiothoracic surgery service in co-ordination with pulmonology and infectious disease service decided surgical approach followed by medical management to be the most favorable treatment plan. This was expected to help both diagnostically and therapeutically, as only the medical management in his case was felt to be inadequate. He underwent inferior vena cava filter placement in preparation for surgery per vascular surgery recommendations. Right thoracotomy followed by decortication and right lower lobectomy was performed. Intraoperatively, after entering the pleura inferiorly, a large abscess cavity was opened revealing liquefied necrosis. Only a small portion of lung tissue was noted to have remained. After the right lower lobe bronchus was transected and stapled, there was some dark blood coming from around the staple line. It was apparent that the basilar artery had “melted” into the bronchus. Further debridement and irrigation of inferior lower lobar abscess was performed. Gross specimen of the right lower lobe of the lungs measured 11.7 x 11.5 x 5.5 cm and weighed 283.6 g. The pleural surface was intact, tan-brown to tan-gray in color, smooth, and soft. No mass lesions or hilar lymph nodes were identified. Pathological reports from lobectomy (Figure 3) revealed pulmonary parenchyma with extensive necrosis, bronchopneumonia, and marked abscess formation. The areas of necrosis were compatible with infarction. However, there was also a marked inflammatory process consisting of neutrophils, eosinophils, lymphocytes, and plasma cells involving the airways and parenchyma. Although vascular intramural inflammatory infiltrates were noted, negative serologies including anti-nuclear antibody and antibodies to myeloperoxidase and serine protease-3 made vasculitis less likely. Pathology was negative for malignancy, and AFB and gram stains were negative for mycobacteria and fungi, respectively. Culture from the lung aspirate grew *Actinomyces odontolyticus*.

The patient improved with treatment and recovered very well from surgery without complications. He was discharged home on oral doxycycline to complete a course of six weeks and apixaban for three months and to be followed up as outpatient.

## Discussion

Pulmonary infarction is usually a complication of another primary disease state, most commonly PE [6]. The infarction can be complicated by secondary infection, thus leading to pulmonary abscess. This can be life-threatening; hence, early diagnosis is key to a better outcome. Since our patient had been healthy without any risk factors, the etiology of his lung lesion was difficult to delineate. Furthermore, the presence of concurrent PE as well as actinomycosis, and the ability of both of these conditions to cavitate made it even more challenging to diagnose. Based on CT findings, we treated the patient for PE and continued further workup to rule out coexisting malignancy versus infectious etiology. In the end, we hypothesized that the DVT was most likely due to apparent trauma in the setting of a hypercoagulable state. DVT was then complicated as PE with infarction, which later cavitated and provided the milieu for lung abscess formation due to secondary infection [7], as suggested by the isolation of *Actinomyces odontolyticus*. On the other hand, one can argue that *Actinomyces* infection is the inciting event causing both a hypercoagulable state and pulmonary cavitary lesion. Indeed, it has previously been reported that pulmonary actinomycosis can present as a cavitary lesion [8]. However, the isolated pulmonary lesion with pathological features of both inflammation and infarction makes this less likely, as the odds for an embolus to lodge only at the exact same site of infection would be relatively low. And, to our knowledge, there are no previous reports on actinomycosis causing pulmonary infarction.

Actinomycosis is a rare and chronic bacterial infection caused by *Actinomyces* species. The symptoms of pulmonary actinomycosis are non-specific and similar to those of other forms of chronic suppurative lung disease. The radiological findings of pulmonary actinomycosis can resemble a broad spectrum of lung pathologies ranging from benign infection to metastases, which makes the disease difficult to diagnose [3]. The disease usually shows peripheral and lower lobe predominance, probably reflecting the role of aspiration in its pathogenesis. In a study, mass-like lesions or consolidations were the most common CT findings. Other radiological findings included cavitation, lymphadenopathy, and ground-glass opacity [9]. *Actinomyces* are noted to form characteristic sulfur granules in infected tissue but not in vitro. The term “sulfur granule” is, however, a misnomer, reflecting only the yellow color of the granule in the pus since the granules are not composed of any sulfur at all. However, not all *Actinomyces* form sulfur granules, e.g., *Actinomyces odontolyticus* [10]. This is why no histological evidence of sulfur granules was seen in our case, even on close re-examination.

The preferred treatment of actinomycosis is high-dose penicillin. Acceptable alternatives are ceftriaxone, doxycycline, or erythromycin. PE should be treated with anticoagulation. Initial therapy is administered as soon as possible in order to quickly achieve therapeutic anticoagulation. Long-term anticoagulant therapy is administered for a period of typically three months (e.g., transient VTE risk factors) or up to 6 to 12 months (e.g. persisting risk factors, unprovoked PE). Selected patients might be candidates for indefinite anticoagulation [10]. Surgery remains an important therapeutic adjunct to pulmonary actinomycosis. It is particularly useful if there are complications, such as well-defined abscesses and empyema, or where discharging fistulas and sinuses may need to be opened up, or, in very rare instances, to control life-threatening hemoptysis. Where surgery has been the initial treatment, even if histology suggests complete resection, it still needs to be followed by prolonged antibiotic therapy, as surgery alone is usually not curative. Inadequate antibiotic therapy postoperatively may result in complications such as bronchopleural fistula and empyema [8].

## Conclusions

We presented a challenging case of cavitary pneumonia with co-existing VTE and *Actinomyces *infection. Both PE and actinomycosis can give rise to cavitary lesions on imaging studies, demanding a high index of suspicion for the diagnosis. Despite that there is no definitive evidence to clearly delineate the process, we believe that the PE and subsequent infarction predisposed to the secondary *Actinomyces* infection. Although rarely reported, actinomycosis and VTE could be an important association. Early recognition and treatment of this association should be embarked to avoid fatal complications.
